# Local pH Effects on the Temperature Dependence of Product Formation in CO_2_ Electrolyzers

**DOI:** 10.1021/jacs.5c20444

**Published:** 2026-01-08

**Authors:** Victor D. Brandão, Oliver Long, Sean Zhong, Rikuto Fushio, Anush Venkataraman, Hakhyeon Song, Marta C. Hatzell, Sankar Nair, Carsten Sievers

**Affiliations:** † School of Chemical & Biomolecular Engineering, 1372 Georgia Institute of Technology, Atlanta, Georgia 30332, United States; ‡ George W. Woodruff School of Mechanical Engineering, Georgia Institute of Technology, Atlanta, Georgia 30332, United States

## Abstract

Large-scale CO_2_ electrolyzers will likely operate at elevated temperatures, but the effect of temperature on the microenvironment near copper catalysts is largely unknown. In this work, we use confocal Raman spectroscopy to reveal that the local pH is a critical parameter controlling product formation during CO_2_ reduction at elevated temperatures. We found that higher temperatures lead to stronger pH gradients consisting of greater surface to bulk pH differences over shorter boundary layers. At −0.6 V, the surface to bulk pH difference at 75 °C was 1.8 units higher than that at 25 °C just from the effect of temperature alone. These results imply that most CO_2_ electrolyzers operating at elevated temperatures were evaluated under much more alkaline microenvironment conditions than previously conjectured at 25 °C. Correlation between surface pH and product analysis shows that a high surface pH (9.9) is beneficial for multicarbon products formation below 45 °C. However, above 55 °C when the surface pH (10.3) becomes too high due to increased surface-bound hydrogen coverage, hydrogenation of C_1_ intermediates is favored, thus compromising carbon–carbon coupling toward C_2+_ products.

## Graphical abstract

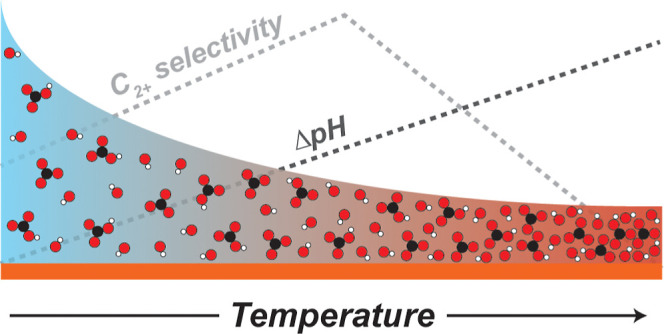

## 1 Introduction

CO_2_ electrolyzers have been extensively studied as a viable technology to convert CO_2_ to multicarbon products.^1,2^ To be competitive with current chemicals manufacturing, the selectivity of multicarbon products needs to be improved.^3,4^ Different microenvironment engineering strategies have been used to modulate the local pH and increase the Faradaic efficiency (FE) of multicarbon products,^5–7^ including tuning the copper electrode hydrophobicity^8^ and adding an inert separation layer on the electrode surface.^9^ In most cases, these strategies are evaluated and benchmarked at room temperature, since near-ambient operating conditions are generally regarded as advantages of electrochemical processes.^10^ However, temperature will be a critical factor in scaling up CO_2_ electrolyzers, since increasing reactor size will change the internal heat distribution,^4,11^ and energy inefficiency and heat generation will inevitably result in electrode heating.^12–14^ Our work shows how temperature regulates the availability of bicarbonate and carbonate and the local pH to more rationally target multicarbon products selectivity.

Local pH has been used as a common descriptor of the reaction microenvironment when evaluating electrolyzer performance.^15^ Local pH refers to the pH near the electrode surface, which is usually units higher compared to the electrolyte bulk pH in mass transport limited electrolyzers.^16^ Different techniques have been used to sample local pH,^17^ including scanning electrochemical microscopy (SECM),^18^ rotating ring-disc electrodes (RRDE),^19^ and optical methods, which generally use pH-sensitive fluorophores to determine pH in membrane-electrode assemblies (MEA).^9^ In situ surface-enhanced infrared adsorption spectroscopy (SEIRAS) has also been used to probe the reaction local pH. Yang et al. used phosphate spectral features to show that CO_2_ reduction at high current densities drives a pH gradient of about 5 pH units between the electrode surface and bulk-phase electrolyte.^16^ Similarly, Ayemoba et al. used bands associated with CO_2_ and bicarbonate to probe local pH over gold electrodes for electrolytes with different cations.^20^ Additionally, surface-enhanced Raman spectroscopy (SERS) has been used to estimate local pH in CO_2_ electrolyzers as a function of potential,^21^ current density, and distance from the electrode surface.^15,22^ Recently, Ma et al. showed how surface pH modulated multicarbon products selectivity for (bi)­carbonate electrolysis for different bulk pH values at 25 °C.^23^ Zhang et al. correlated increasing temperature with higher local pH and lower H_2_ FE at 50 mA/cm^2^ over a silver foam,^15^ but this observation disagrees with most studies reporting higher H_2_ FE at higher temperatures on copper.^10,24,25^ While these studies have analyzed the effects of different parameters on local pH, a comprehensive study on how temperature impacts the distribution of species near the catalyst surface and, thereby, product formation at varying potentials is still lacking.

Some studies have conjectured about the effect of temperature on the reaction microenvironment based on product distribution trends alone. On one hand, moderately high local pH was found to benefit ethylene formation and inhibit hydrogen and methane evolution.^26,27^ On the other hand, excessively high local pH was found to favor the reaction between CO_2_ and hydroxide anions to form bicarbonate,^28,29^ thus lowering in situ-generated CO_2_, sacrificing ethylene FE, and promoting hydrogen evolution.^26,30^ To avoid issues associated with low CO_2_ supply near the catalyst under high local pH conditions, gas diffusion electrodes (GDE) have been used to enable direct delivery of CO_2_ at high concentrations to the catalyst surface by separating electrolyte and gas feed streams.^31^ Ahn et al. suggested that the concentration of dissolved CO_2_, rather than the pH, is the determinant factor controlling product formation between 2 and 42 °C.^25^ Vos et al. argued that local pH is one of the factors regulating product distribution between 18 and 48 °C and that very high local pH controls product distribution between 48 and 70 °C.^10^ Neither of these studies provided quantitative insight into the extent to which temperature affects local pH and surface intermediate coverage, which is what ultimately controls product formation. While higher temperatures will lead to higher current densities^32^ and, consequently, greater consumption of protons resulting in increased local pH, the extent with which local pH increases with temperature, the impact of that on the surface mechanism regulating product formation, and the pH threshold determining coverage trade-off and maximum multicarbon product selectivity remain unclear.

In this study, we coupled confocal Raman spectroscopy with product analysis to investigate the local concentrations of bicarbonate and carbonate and the local pH as a function of cathodic potential (0.1 to −0.6 V), temperature (25 to 75 °C), and distance from the electrode surface. We correlated changes in local pH with product selectivity to provide a complete picture of how temperature regulates the reaction microenvironment by controlling the distribution of species near the electrode surface. Our work suggests that local pH is a critical parameter regulating the product distribution dependence on temperature under practical CO_2_ reduction conditions.

## 2 Results and Discussion

### 2.1 Bicarbonate and Carbonate Equilibrium and Surface pH

The near-surface concentrations of bicarbonate and carbonate anions in the reaction microenvironment were probed experimentally (Section S1) as they are directly influenced by polarization of the copper electrode between 0.1 and −0.6 V. Lower current densities were used to prevent resistive heating and temperature drifting of the electrode during experiments. Resistive heating at industrially relevant current densities has been shown to cause electrode surface temperature variations of more than 10 °C,^14,32^ and even a 10 °C temperature variation can significantly affect surface CO population^33^ and the reaction microenvironment. Two bands centered at 1015 and 1065 cm^–1^, which are attributed to solution-phase bicarbonate and carbonate, respectively,^34–36^ were used to quantify the local mole fractions of these species. Figure 1a illustrates the evolution of these bands at 25 °C, and Figure S2 shows these bands at all other temperatures. More negative potentials led to a decrease in the bicarbonate band and an increase in the carbonate band at all temperatures between 25 and 75 °C. Control experiments were performed to correlate the ratio between the area of these two bands and the ratio of their local concentrations (Figure S3). Bicarbonate and carbonate local concentrations and surface pH were then calculated from this latter ratio, as described in the Supporting Information (Section S2). Figure 1b shows the effect of potential and temperature on the mole fractions of bicarbonate and carbonate. At 25 °C, decreasing the potential from 0.1 to −0.6 V led to a slight decrease (1.1 percentage points) in bicarbonate concentration and an increase in carbonate concentration, which became more evident at higher temperatures.

**Figure 1:**
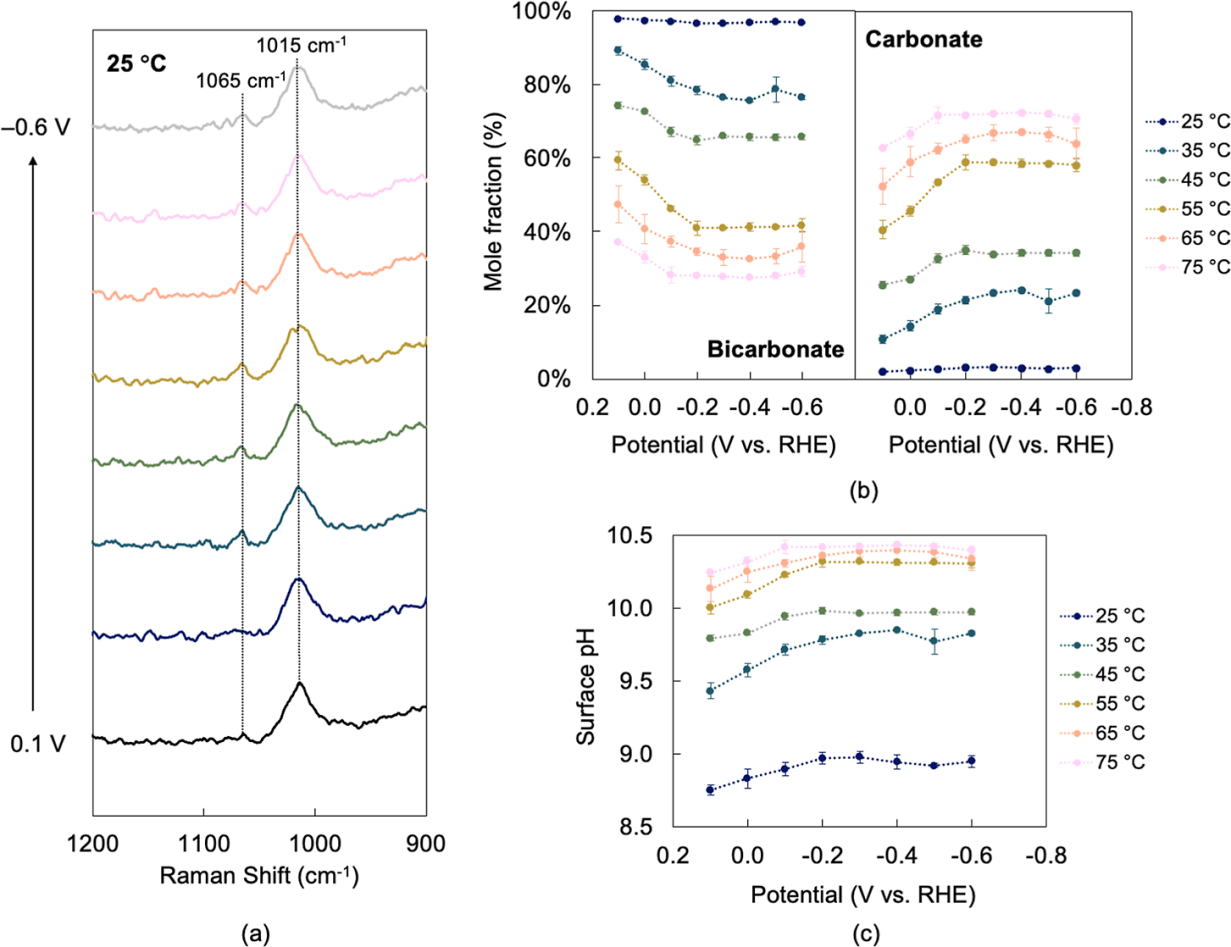
Abundance of bicarbonate and carbonate anions near the electrode surface. (a) Representative spectra at 25 °C showing the evolution of bicarbonate and carbonate bands centered at 1015 and 1065 cm^–1^, respectively. (b) Mole fractions of anions show decreasing bicarbonate and increasing carbonate concentrations at more negative potentials and higher temperatures. (c) Surface pH increases at more negative potentials and higher temperatures.

In alkaline environments, more negative potentials drive the consumption of bicarbonate through two different paths: formation of in situ CO_2_ followed by its reductive adsorption (eq 1), which is known to be the active intermediate mediating carbon reduction on the surface,^37^ and conversion to carbonate (eq 2), which increases the local concentration of carbonate and provides a hydrogen source (H_2_O) for proton–electron transfer steps.

1 HCO3−+e−+∗⇌C*O2−+OH−

2 $$HCO_{3}^{-}+OH^{-}⇌CO_{3}^{2-}+H_{2}O$$

Higher temperatures led to lower fractions of bicarbonate and higher fractions of carbonate for a given potential. At −0.6 V, the mole fraction of bicarbonate decreased by 67.7 percentage points between 25 and 75 °C. Increasing temperature favored the conversion of bicarbonate via eqs 1 and 2, raising alkalinity and locally producing more H_2_O. Furthermore, increasing temperature did not seem to have a dramatic effect on the dependence of local concentrations on potential. At 75 °C, decreasing the potential from 0.1 to −0.6 V led to a 7.9 percentage point mole fraction change, which is slightly above the one observed at 25 °C.

Surface pH increased at more negative potentials for a given temperature (Figure 1c). At 25 °C, the surface pH increased from 8.8 at 0.1 V to 9.0 at −0.6 V. Two potential windows can be identified with different surface pH variations at all temperatures: one between 0.1 V and −0.2 V, where the pH slightly increased, and one between −0.2 V and −0.6 V, where it remains virtually constant. Between 0.1 and −0.2 V, the surface pH increase should be a direct consequence of the accumulation of hydroxide anions produced from eq 1 and from the reductive adsorption of water molecules formed in eq 2, as represented in eq 3 (Volmer step).

3 $$H_{2}O+e^{-}+*⇌H_{ad}+OH^{-}$$

Between −0.2 and −0.6 V, the relatively constant surface pH suggests that the rate of hydroxide anion consumption in eq 2 is approximately equal to its production rate in eqs 1 and 3. In this potential window, the surface pH remained practically unchanged at all temperatures, suggesting that the formation and consumption of bicarbonate and carbonate reached dynamic equilibrium. In particular, carbonate reacts with water to generate bicarbonate (reverse of eq 2) under these conditions, since increasing cathodic current actively depletes near-surface bicarbonate according to eq 1. While bicarbonate replenishment from the bulk solution is also an active bicarbonate source, our observations indicate that the conversion of carbonate through eq 2 is the main bicarbonate source near the catalyst surface. Figure S4 shows how the absolute areas for the Raman bands associated with bicarbonate and carbonate vary as a function of potential at 35 °C. Between 0.1 and −0.2 V, CO_2_RR current promotes bicarbonate consumption and generates hydroxide anions (eq 1), thus leading to bicarbonate band area decrease. As the current is still reasonably low, hydroxide further reacts with bicarbonate to give carbonate (eq 2), thus leading to the observed increase in the carbonate band area. Between −0.2 and −0.6 V, increased CO_2_RR current consumes bicarbonate more significantly via eq 1. This shifts the equilibrium in eq 2 to promote the conversion of carbonate to bicarbonate. The band areas associated with both anions decrease (Figure S4). As they decrease by roughly the same amount, the mole fractions of these anions remain generally unchanged, thus suggesting that their formation and consumption reached equilibrium.

Higher temperatures led to higher surface pH values, which reached 10.4 at 75 °C and −0.6 V, reflecting an approximate 1.4 pH units increase from its value at 25 °C. Figure 2 shows the difference between surface pH and electrolyte bulk pH (ΔpH) as a function of temperature at −0.6 V. Trends at other potentials are shown in Figure S7. We highlight that the electrolyte bulk pH slightly decreases with increasing temperature (Table S2). For all temperatures between 25 and 75 °C, the surface pH was higher than bulk pH, leading to positive ΔpH differences. Furthermore, ΔpH increased with increasing temperature from 0.57 at 25 °C to 2.16 at 55 °C and 2.35 at 75 °C.

**Figure 2:**
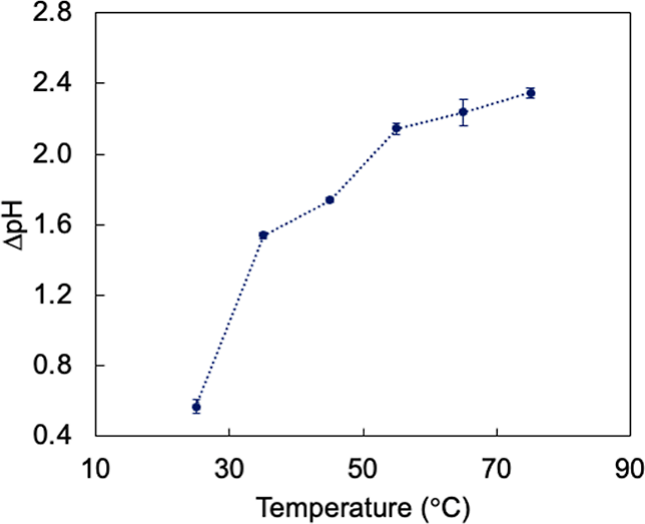
Difference between surface pH and electrolyte bulk pH (ΔpH) at −0.6 V indicates an increase of 1.8 pH units between 25 and 75 °C just from the effect of temperature alone. Trends at other potentials are shown in Figure S7.

We highlight that an inverse trend of decreasing surface pH with more negative potentials is observed if electrodes are not reduced at −0.7 V prior to the cathodic sweep. This is due to the formation of Cu_2_(OH)_2_CO_3_ (malachite),^21^ an insoluble salt whose formation removes hydroxide anions from solution, thereby resulting in pH decrease. The trend has been analyzed at 25 °C and is described in detail in the Supporting Information (Section S4, Figure S8). To further confirm the absence of malachite in our study, copper electrodes were characterized before and after reaction at 25 °C (Section S5, Figures S9, S10, S11). We found that copper was mostly present as copper metal and not as Cu^2+^ and that the electrode surface was considerably smoother after the reaction.

While previous studies have conjectured the effect of temperature on local pH,^10,25^ they have not thoroughly assessed how dramatic this effect would be. We show that pH gradients in CO_2_ electrolyzers can be as high as 1.8 pH units just from the effect of temperature build-up alone. In practical terms, CO_2_ electrolyzers will likely operate under much more alkaline microenvironment conditions than previously conjectured at 25 °C.^16^ Greater pH gradients across the microenvironment suggest that increasing temperature favors the local consumption of water as a hydrogen donor and, subsequently, the accumulation of hydroxide anions close to the electrode surface. In fact, previous theoretical calculations have established that more hydroxide anions can be found near the electrode surface as temperature increases.^38^ This significant increase in surface pH indicates that the local availabilities of water and hydroxide anions directly regulate the product formation dynamics across the reaction boundary layer at different temperatures.

### 2.2 Ion Transport and Boundary Layer

The concentration profiles of bicarbonate and carbonate in the reaction boundary layer were determined by moving the focal plane of spectra collection to different distances from the electrode surface. While diminished plasmonic enhancement from the metal surface caused the intensities of the bicarbonate and carbonate bands to decrease, local pH could still be profiled across the reaction microenvironment. Figure 3a shows how the pH varied with distance from the electrode surface at −0.6 V for different temperatures. At all temperatures, the pH decreased from its surface value (at *z* = 0) to its bulk value at varying distances from the electrode surface. At 25 °C, the pH decreased from 8.9 to its bulk value (8.4) in 1200 μm. While previous studies have estimated thinner boundary layers (250 μm) above gas diffusion electrodes (GDEs),^15,23^ we highlight that different CO_2_ delivery mechanisms and variable shear forces associated with different electrolyte flow rates directly impact ion transport and mass transfer resistances, which could thereby have contributed to thinner boundary layers.

**Figure 3:**
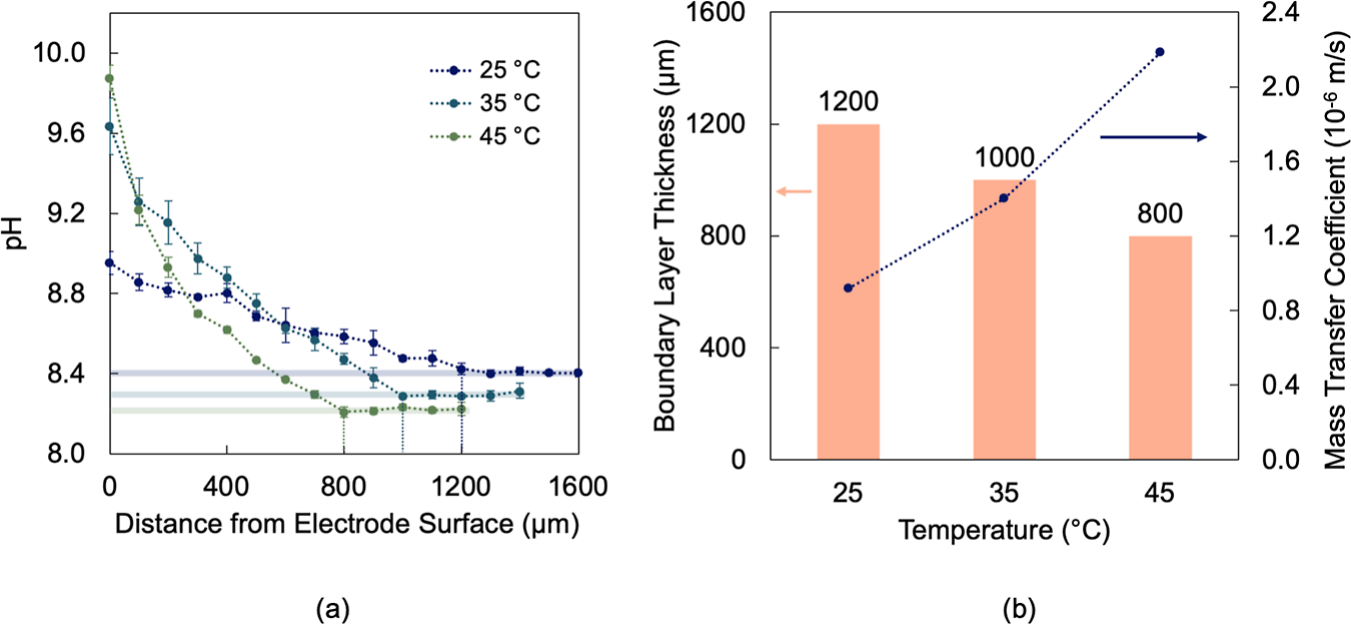
pH gradient across the reaction microenvironment at −0.6 V. (a) pH dependence on distance from the electrode surface at different temperatures. (b) Boundary layer thickness and bicarbonate mass transfer coefficients as a function of temperature.

Higher temperatures led to greater differences between surface and bulk pH but shorter distances to reach bulk pH, i.e., thinner boundary layers. Figure 3b shows an approximate 400 μm thickness reduction between 25 and 45 °C at −0.6 V. Thinner boundary layers are expected at higher temperatures due to faster diffusion of ionic species. To quantify this trend, mass transfer coefficients for the bicarbonate anion were calculated based on its temperature-dependent diffusivity and the experimentally determined boundary layer thickness. Detailed calculations are described in the Supporting Information (Section S6). The transfer coefficient increased from 0.92 × 10^–6^ m/s at 25 °C to 2.18 × 10^–6^ m/s at 45 °C, indicating that the bicarbonate anion diffusion rate practically doubled (Figure 3b). This corresponded to a Sherwood number increase from 8.3 to 12.5. Higher current densities were observed at higher temperatures, which implies faster formation and desorption of reaction products near the surface, thus contributing to enhanced local mixing, increased convection transport, and higher Sherwood numbers. While ion transport and boundary layer formation are closely related to reactor architecture and should be different for other electrolyzer systems, the underlying impact of temperature in increasing the surface to bulk pH difference and reducing the boundary layer thickness, i.e., in intensifying the pH gradient, is a mechanistic implication that holds true for any system.

### 2.3 Product Formation and Microenvironment Dynamics

Local alkalinity regulates intermediate conversion and product formation as a function of temperature between 25 and 75 °C. The main products formed during CO_2_ electrolysis at −0.6 V with nonnegligible Faradaic efficiencies were hydrogen, carbon monoxide, methane, ethylene, formic acid, and ethanol. Faradaic efficiencies for each product are indicated in Figure S12. Figure 4a shows the accumulated Faradaic efficiencies for monocarbon (C_1_), multicarbon (C_2+_) products, and hydrogen as a function of temperature at −0.6 V. Hydrogen Faradaic efficiency increased by 15.1 percentage points between 25 and 65 °C, which agrees with previous studies reporting the dominance of the hydrogen evolution reaction at higher temperatures.^10,24,25^ Among carbon products, monocarbon products were the major products formed regardless of temperature, and their selectivity decreased by 6.3 percentage points between 25 and 65 °C. Multicarbon products selectivity increased by 2.9 percentage points between 25 and 45 °C and decreased by 4.3 percentage points between 45 and 65 °C. This corresponded to a maximum C_2+_ to C_1_ FE ratio of about 0.49 at 45 °C. A similar maximum in C_2+_ products FE at around 45 °C has been observed in previous studies using bicarbonate electrolytes.^10,39,40^ In our previous work, we showed that adsorbed CO (CO_ad_) coverage on defect sites is also maximized at 45 °C.^33^ A similar Faradaic efficiency increase for ethylene was observed between 20 and 40 °C in an MEA electrolyzer operating at higher current densities between 50 and 200 mA/cm^2^ (Figure S13). This suggests that the underlying variable controlling the dependence of product formation on temperature is not directly the current density itself but the availability of the hydrogen source near the catalyst surface. Interestingly, the difference between surface and bulk pH increased most dramatically between 25 and 55 °C at −0.6 V (Figure 2), which coincides with the temperature interval in which multicarbon product selectivity increased to a maximum. ΔpH increased by approximately 1.6 units between 25 and 55 °C, suggesting that raising the temperature increased the local consumption of water near the surface, thereby leading to hydroxide accumulation and increased alkalinity gradient. Above 55 °C, ΔpH remained virtually unchanged and surface pH seemed to plateau. Since local carbonate anions and the bulk electrolyte can readily replenish bicarbonate near the surface, we do not expect bicarbonate to be the reactant limiting hydroxide formation. Rather, we attribute the less pronounced pH gradient increases above 55 °C to the limited availability of surface sites to keep adsorbing H_ad_ at higher temperatures. At higher temperatures, the rate with which H_ad_ are replenished on the surface is likely higher than the rate with which they couple to desorb as H_2_ gas. This results in a surface with a high concentration of H_ad_ as the most abundant surface species, thus hindering further water deprotonation and hydroxide generation (eq 3). The change in extent with which hydroxide is generated and water is consumed indicates that local alkalinity likely regulates intermediate conversion and product formation on the surface.

**Figure 4:**
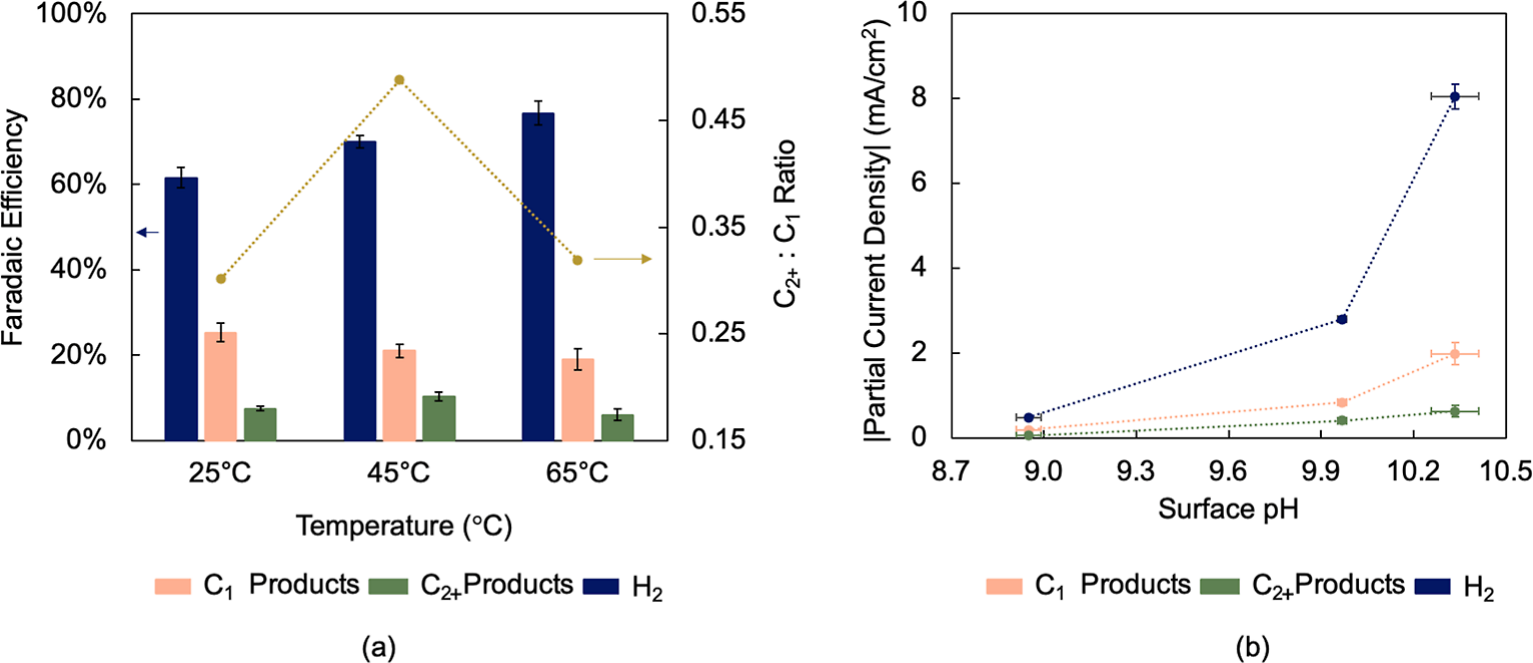
Product formation dependence on temperature and surface pH at −0.6 V. (a) Faradaic efficiencies and C_2+_ to C_1_ products ratio as a function of temperature. Faradaic efficiencies for each individual product are indicated in Figure S12. (b) Product current densities as a function of surface pH.

To better understand the comparative relevance of these local pH gradients to the temperature dependence of product formation, other temperature-dependent variables in the system were studied. Electrochemical impedance spectroscopy (EIS) was used to evaluate the electrolyte resistance between 25 and 75 °C at −0.6 V. The associated Nyquist plots revealed a decrease of the electrolyte resistance at higher temperatures (Figure S14a). The decrease at −0.6 V was similar to the one observed at open circuit potential (OCP) and reflected a marginal 34% reduction from 2.24 to 1.49 Ω (Figure S14b). Thus, electrolyte resistance effects are minimal. Additionally, the CO_2_ solubility was calculated as a function of temperature based on Carroll et al.’s correlation for Henry’s constant (Figure S14c). The solubility decreased by 61% from 34.65 to 13.53 mM. Since the observed current densities were below 10 mA/cm^2^ (Figure 4b), CO_2_ conversion was minimal, and supply was plentiful. Thus, the effects of CO_2_ solubility are also minimal. Furthermore, we used surface-enhanced infrared spectroscopy to follow the CO_ad_ band between 2150 and 1800 cm^–1^ (Figure S15) and showed that CO coverage increases between 25 and 45 °C (13%) and decreases between 45 and 75 °C (65%) (Figure S14d).^33^ This trend closely resembles the C_2+_ product selectivity trend in Figure 4a and is associated with a local pH gradient increase of 312% between 25 and 75 °C, a much more significant figure compared to those of the other effects listed. This increase is a result of hydrogen source consumption and hydroxide accumulation. The rate of consumption of the hydrogen source affects the coverage of H_ad_ and CO_ad_, which compete for surface sites. Thus, local pH and CO adsorption are intrinsically related effects and the main drivers of the product distribution dependence on temperature.

To evaluate the kinetics of product formation, H_2_, C_1_, and C_2+_ product current densities were studied as a function of surface pH (Figure 4b). The formation rates of H_2_ and C_1_ products were greater than that of C_2+_ products for any given surface pH. Higher current densities were observed with increasing surface pH for all products since the formation of any product requires H_ad_ consumption, thus resulting in H^+^ depletion and OH^–^ buildup (Figure S16a,b). The extent to which each current density increased with temperature, however, was dramatically different depending on the product formed. On one hand, H_2_ current density increased from 0.49 mA/cm^2^ at pH 8.95 to 8.05 mA/cm^2^ at pH 10.34, which corresponds to an increase by a factor of 16.4 (Figure 4b). On the other hand, C_2+_ product current density increased from 0.06 to 0.64 mA/cm^2^ for the same pH difference, an increase by a factor of 10.7. Thus, while microenvironment engineering strategies to increase local pH will lead to higher product formation rates, the weaker dependence of multicarbon current density on local pH suggests that these will be formed with lower selectivity. Previous work by Hori et al. showed that changing the bulk pH in CO electroreduction does not affect the formation rates of C_2+_ products^41^ since the associated rate-determining step is a proton-independent CO_ad_–CO_ad_ coupling reaction.^42,43^ Local pH is a critical parameter controlling interfacial processes responsible for C_2+_ product formation and selectivities^27^ as it directly affects bicarbonate conversion to CO_ad_, CO_ad_ coverage, and CO_ad_–CO_ad_ coupling depending on surface site competition with adsorbed hydrogen species. Multiple studies have further demonstrated the critical role local pH plays in determining the C_2+_ product distribution.^44–47^

The observed local pH and product distribution trends are direct consequences of competing H and CO adsorption. Scheme 1 illustrates how increasing the temperature changes the reaction microenvironment, thus resulting in these trends. Higher temperatures increase the CO_2_ reduction current density, which converts more local bicarbonate to in situ CO_2_, producing more hydroxide anions (eq 1) and raising surface pH. Thermodynamically, higher temperatures also favor the deprotonation of bicarbonate to carbonate (Figure S5) and decrease CO_2_ solubility in the bulk electrolyte (Figure S14c). In alkaline media, this leads to the local formation of water (eq 2). As a result, the reductive adsorption of water is favored, forming surface-bound hydrogen (H_ad_) (eq 3). At higher temperatures, surface diffusion rates of both CO_ad_ and H_ad_ increase,^38^ and, as H_ad_ coverage increases, surface-bound hydrogen and CO_ad_ compete for surface sites. While H_ad_ can partially displace CO_ad_, CO_ad_ is unable to displace H_ad_ to any extent.^48^ Furthermore, linearly bound CO_ad_ coverage increases between 25 and 45 °C^49^ and decreases above 45 °C,^33^ as highlighted in Scheme 1. Together with higher surface pH values, this provides evidence that H_ad_ coverage is likely higher than CO_ad_ coverage at higher temperatures. H_ad_ coupling (eq 4, Tafel step) or reaction with another water molecule (eq 5, Heyrovsky step)^50^ will promote increased hydrogen gas formation, as observed at higher temperatures in Figure 4a.

4 $$H_{ad}+H_{ad}⇌H_{2}$$

5 $$H_{ad}+e^{-}+H_{2}O⇌H_{2}+OH^{-}$$

**Scheme 1:**
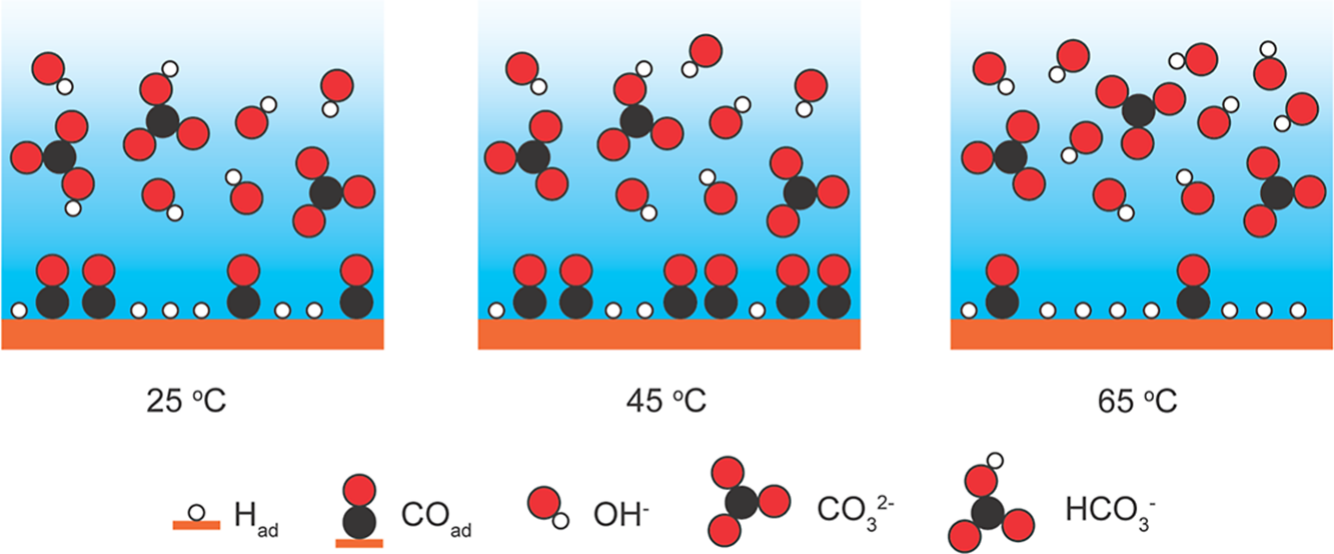
Schematic of Dynamic Microenvironment Changes as a Function of Temperature^a^

a At higher temperatures, bicarbonate deprotonates to carbonate, and the microenvironment becomes richer in hydroxide anions. On the surface, CO_ad_ coverage increases between 25 and 45 °C and H_ad_ coverage becomes dominant above 55 °C.

It has been well established that OH^–^ anions do not promote C–C coupling and C_2+_ products formation.^51^ Previous work by Ma et al. showed that while OH^–^ anions do not facilitate carbon–carbon coupling or affect total C_2+_ formation, they influence the distribution of C_2+_ products since higher OH^–^ concentrations facilitate acetate formation via homogeneous reactions while correspondingly suppressing other C_2+_ products such as ethylene, ethanol, and *n*-propanol.^52^ Our results show that hydroxide accumulation is an observable result of increased local water deprotonation at higher temperatures, which directly impacts the competition of CO_ad_ and H_ad_ for surface sites. The relative coverages of CO_ad_ and H_ad_ are the critical variables controlling the effect of temperature on C_1_ and C_2+_ product formation pathways.

The increasing ΔpH trend between 25 and 55 °C (Figure 2) suggests that the formation of adsorbed hydrogen leading to hydrogen gas evolution and alkalinity increase intensifies in this temperature range. HER is limited by how well H_ad_ can displace CO_ad_ to populate the copper surface, and increases in pH primarily reflect increased hydrogen donor (H_2_O) consumption and H_ad_ formation. In this case, hydrogen gas is equally likely to be formed via eq 4 (direct H_ad_ coupling) or eq 5 (reaction between H_ad_ and H_2_O).^50^ Above 55 °C, virtually constant surface pH values indicate that the production of hydroxide anions does not further increase, reflecting that water deprotonation is limited by the number of sites on which hydrogen can adsorb. At this point, the surface probably reached its maximum H_ad_ coverage. Since the production of hydrogen gas keeps increasing above 55 °C, this indicates that H_ad_ coupling via eq 4 is likely the most dominant surface process at higher temperatures. Thus, the sharp increase in local pH followed by a less pronounced pH increase above 55 °C is influenced by the dominant HER pathway controlling hydrogen consumption, which is directly affected by the availability of H_ad_, and thus by the extent with which water is consumed.

Adsorbed hydrogen can also be used in proton–electron transfer steps during CO_2_ reduction. As a matter of fact, methane and H_2_ formation were shown to be linked through a common H_ad_ intermediate as CH_4_ is formed by the reaction between CO_ad_ and H_ad_ and not CO_ad_ and solution-phase H^+^.^53^ While some increase in H_ad_ coverage—up to 45 °C, when ΔpH is 1.7 and surface pH is 9.9—seems to be beneficial for C_2+_ products formation since it guarantees a H_ad_ pool for proton–electron transfer steps, excessive H_ad_ coverage—above 45 °C—directly leads to HER and hydrogenation of adsorbed C_1_ intermediates. This hinders CO–CO and/or CO–COH coupling pathways toward multicarbon products, since these would require a sufficiently high density of adjacent CO_ad_ species. Under these conditions, hydrogen coupling and CO_ad_ hydrogenation routes toward monocarbon oxygenated intermediates will likely be the dominant reaction pathways at higher temperatures.

## 3 Conclusions

In this work, we investigated the effect of temperature on the local concentrations of bicarbonate and carbonate, local pH, and product formation at different cathodic potentials. Higher temperatures led to stronger pH gradients, which consisted of greater surface to bulk pH differences over shorter reaction boundary layers. This implies that CO_2_ electrolysis studies carried out at higher temperatures were likely evaluated under much more alkaline microenvironment conditions than those previously conjectured at room temperature. The increase in local pH was particularly sharp between 25 and 55 °C, when a maximum in C_2+_ product selectivity was observed. By correlating local pH measurements with product selectivities, we showed how temperature affects CO_ad_ and H_ad_ coverages and, thus, regulates product formation pathways on the catalyst surface. While previous studies have used local pH changes to reason temperature-dependent observations,^10,25^ these have just been conjectured from product selectivity data and have not been explained in detail from a mechanistic perspective. This work gives direct spectroscopic evidence that surface pH values below 9.9 are beneficial for C_2+_ products formation by ensuring hydrogen is supplied to proton–electron transfer reactions below 45 °C. We also show that excessively high surface pH values above 10.3 result from increased near-surface water deprotonation above 55 °C, which reduces CO_ad_ coverage, compromising carbon–carbon coupling and favoring CO_ad_ hydrogenation and hydrogen evolution.

Quantitative local pH analysis, which was carried out here by controlling reaction temperature, highlights how surface pH is a critical parameter driving the dependence of product distribution on temperature and helps better inform pH ranges to target the production of mono- and multicarbon products. Without adequate thermal management, practical CO_2_ electrolyzers will likely operate in local microenvironments that are dramatically different from those at 25 °C. While different reactor designs and operating conditions might change the concentration profiles of bicarbonate and carbonate and, therefore, the numerical values of pH, this work provides system-agnostic understanding of the extent with which temperature impacts microenvironment composition and pH gradients regulating surface phenomena on polycrystalline copper electrodes. In that sense, using temperature to control the microenvironment in electrolyzers could be a useful strategy to target multicarbon products synthesis in practical large-scale applications.

## Supplementary Material



## Notes

Experimental methods, local pH calculations, species distribution, malachite formation, electrode characterization, mass transfer calculations, experimental setup, supporting Raman spectra, bicarbonate and carbonate band areas, calibration curve for concentrations vs band ratios, van’t Hoff plots for ionization constants, ΔpH as a function of potential, SEM images, EDS analysis, AFM profiles, XPS spectra, Faradaic efficiencies of individual products, Faradaic efficiencies of ethylene at high current densities for MEA electrolysis, EIS plots, CO_2_ solubility curve, CO coverage profile, carbonic acid ionization constants, and electrolyte bulk pH values (PDF)
