# Supplementary material for: Local pH Effects on the Temperature Dependence of Product Formation in CO2 Electrolyzers
Source: J Am Chem Soc. 2026 Jan 8;148(2):2801–9. doi: 10.1021/jacs.5c20444 (PMC12833869; doi:10.1021/jacs.5c20444)
Supplement: Supplementary file 1 [file ja5c20444_si_001.pdf]

Supporting Information

for

Local pH Effects on the Temperature Dependence of Product Formation in CO_2 Electrolyzers

Victor D. Brandão[a], Oliver Long[a], Sean Zhong[a], Rikuto Fushio[a], Anush Venkataraman[a], Hakhyeon Song[b], Marta C. Hatzell[b], Sankar Nair[a], Carsten Sievers[a,*].

[a]School of Chemical & Biomolecular Engineering, Georgia Institute of Technology, Atlanta, Georgia, 30332, USA

[b]George W. Woodruff School of Mechanical Engineering, Georgia Institute of Technology, Atlanta, Georgia, 30332, USA

* Corresponding author's email address: carsten.sievers@chbe.gatech.edu

Supporting Discussion

S1. Experimental Section

S1.1. Materials

Copper foils (99.999%) from Sigma-Aldrich were used as electrode precursors. Alumina suspensions (1.0 μm and 0.3 μm) from Electron Microcopy Sciences (EMS) were used to mechanically polish the copper electrodes. H_3PO_4 (85%) was purchased from J. T. Baker and $KHCO_3$ (> 99.95%) was purchased from Sigma-Aldrich. Electrolytes were prepared with Milli-Q water (> 18.2 MΩ) and cleaned with Chelex (100 sodium form) prior to experiments. Anion exchange membranes (Sustainion X37-50 Grade RT) were purchased from Dioxide Materials. Argon (Ultra High Purity) and CO_2 (Instrument Grade) from Airgas were used to purge the electrolyte.

S1.2. Electrode Preparation and Characterization

Copper foils were sequentially polished in 1.0 and 0.3 μm alumina suspensions followed by electrooxidation in 10% H_3PO_4 for 2 minutes at 1.6 V (vs. RHE) against a graphite counter electrode. Subsequently, electrodes were reduced in 1.0 M $KHCO_3$ for 20 minutes at –0.7 V (vs. RHE) to ensure copper was fully reduced on the surface exposed to the electrolyte. Data for replicates were collected on different copper electrodes independently prepared following this procedure.

Electrodes were characterized before and after reaction without any further sample preparation steps. Scanning electron microscopy (SEM, Thermo Axia Variable Pressure SEM) coupled with energy-dispersive spectroscopy (EDS) mapping was used to sample the electrode morphology and atomic composition. Spectra were collected with a 5-mm spot size and 5-kV acceleration voltage at a 10-mm working distance. X-ray photoelectron spectroscopy (XPS,

Thermo K-Alpha) was used to probe copper oxidation states on the surface. XPS spectra were collected on a 400-μm spot with a pass energy of 50 eV and an energy step size of 0.1 eV with the coaddition of 10 consecutive scans. Atomic force microscopy (AFM, Bruker Icon) was used to sample the surface topography and roughness. AFM profiles were collected in tapping mode with an RTESPA-300 tip (Bruker) using a 10-μm scan size and 256 samples per line at a 0.4-Hz scan rate and 30-mV drive amplitude.

S1.3. Electrochemical Methods

Copper electrodes were assembled into a custom-built two-compartment spectroelectrochemical cell (**Figure S1**). Prior to experiments, the cell was cleaned with a concentrated H_2SO_4/HNO_3 solution and sonicated in Milli-Q water. Experiments were performed in 1.0 M $KHCO_3$ using a three-electrode configuration with a platinum counter electrode and a saturated Ag/AgCl reference electrode. Potential was controlled using a one-channel potentiostat (1010B, Gamry) and is reported in the reversible hydrogen scale (RHE) using $E_{RHE} = E_{Ag/AgCl} + 0.197 + \frac{R \cdot T}{F \cdot \log e} pH$. Electrolyte resistance was evaluated as a function of temperature with potentiostatic electrochemical impedance spectroscopy (EIS). Measurements were carried out in the 20 kHz – 200 mHz frequency range with a sine amplitude of 10 mV and 10 points/decade for each temperature at open circuit potential (OCP) and at –0.6 V. Spectra were fitted using an equivalent circuit model with a constant phase element (CPE) to account for non-idealities of the double layer capacitance. Electrolyte resistance was numerically determined from the model best fit through the first intersection of the semi-circle with the real impedance axis. 90% ohmic compensation was performed for iR_u correction. Cell temperature was controlled using a temperature probe inside the cell and a heating element connected to a temperature controller (Applied Test Systems). The electrode temperature was measured with an IR thermometer (Kizen,

LaserPro LP220) prior to experiments to ensure stable temperature reading. A mass flow controller (Omega, FMA) was used to deliver argon and CO_2 to the cell. Prior to experiments, argon was purged at 50 sccm for 20 minutes to minimize atmospheric oxygen contamination, and CO_2 was purged at 50 sccm for 20 minutes to saturate the electrolyte. CO_2 bubbling was the only source of agitation inside the cell during experiments. Gas and liquid products were collected after 20-minute electrolysis at a given potential and temperature. Gas products (H_2, CO, CH_4 and C_2H_4) were quantified using gas chromatography (490 μGC, Agilent) equipped with 3 columns (Molesieve 5Å/Ar, Molesieve 5Å/He, and Poraplot U/He) and a thermal conductivity detector (TCD). Liquid products (HCOOH and H_3CCH_2OH) were quantified using high-performance liquid chromatography (HPLC, Shimadzu) with an Aminex HPX-87H column (Bio-Rad) and a UV/visible (UV/vis) detector and a refractive index detector (RID). Faradaic efficiencies (FE) were calculated using $FE = znF/iAt$, where z is the number of electrons (2 for H_2, CO and HCOOH, 8 for CH_4, and 12 for C_2H_4 and H_3CCH_2OH), n is the molar quantity of product detected, F is Faraday's constant (F = 96485 C/mol), i is the current density (A/cm^2), A is the electrode geometric area (cm^2), and t is the electrolysis time (s). Results are presented as an average of three independent experiments.

S1.4. MEA Experiments

A membrane-electrode assembly (MEA, Scribner) electrolyzer was used to perform CO_2 electrolysis at high current densities between 50 and 200 mA/cm^2. The experimental setup was described in detail by Song et al.[1] A 5 cm^2 copper electrode was used as cathode and a 5 cm^2 nickel foam (MSE Supplies) was used as anode. A bipolar membrane (BPM, Fumasep FMB) was used as separating media between cathode and anode. 100 mL of a 5.0 M potassium carbonate solution was circulated as catholyte in an interdigitated flow plate at a flow rate of 65 ml/min by a peristaltic

pump (Masterflex L/S pump). 200 mL of 1.0 M potassium hydroxide was circulated as anolyte at 130 mL/min. Gas and liquid products were collected after 1-hour electrolysis at a given current density and temperature.

S1.5. SERS Experiments

In-situ surface-enhanced Raman spectroscopy (SERS) was used to probe local concentrations of species between 0.1 and –0.6 V at temperatures between 20 and 80 °C. Spectra were collected with a confocal Raman spectrometer (inVia, Renishaw) and a 5x objective (Leica) using a 785 nm laser at ~ 30 mW power and 1200 lines/mm grating. Spectra are the coaddition of 4 accumulations, each at a 10-second acquisition time on a given spot on the copper surface. Representative Raman bands were baseline-corrected, and Gaussian fits were used to calculate their areas. Results are reported as an average of band areas calculated for spectra taken for at least three independent experiments and/or spots on the surface to circumvent the effect of surface bubbles and differences in local enhancement.

S2. Local pH Calculation

Control experiments were performed to correlate the local concentrations of bicarbonate and carbonate anions (p) with their band areas ratio. **Figure S2** shows that the concentration ratio p is 4.53 greater than the band area ratio:

$$p = \frac{[HCO_3^-]}{[CO_3^{2-}]} = 4.53 \cdot \frac{I_{HCO_3}^{-}}{I_{CO_3^{2-}}} \quad \textbf{(S1)}$$

While band intensities varied with experimental parameters, including laser power, sample exposure time and local enhancement factors, the ratio of bands was experiment agnostic. At pH > 8, the equilibrium concentrations of CO_2 (aq) and H_2CO_3 are negligible at all temperatures between 25 and 75 °C (**Figure S5**). Hence, bicarbonate and carbonate are the only contributing species at equilibrium and their percent mole fractions can be calculated as:

$$x_{HCO_3^-} = 100 \cdot \frac{p}{1+p} \quad \textbf{(S2)}$$

$$x_{CO_3^{2-}} = 100 \cdot \frac{1}{1+p} \quad \textbf{(S3)}$$

These expressions were used to plot the percent mole fractions in **Figure 1b.** The species distributions in **Figure S5** were calculated according to **Section S3** using pK_{a1} and pK_{a2} values from **Table S1**, which were derived from Harned et al.'s ionization constants between 0 and 50 °C (**Figure S6**).[2, 3] The local concentration of H^+ was calculated from the expression for pK_{a2}:

$$K_{a2} = \frac{[H^+][CO_3^{2-}]}{[HCO_3^{2-}]} \rightarrow [H^+] = K_{a2} \cdot p \quad \textbf{(S4)}$$

Local pH values were then calculated with the following expression:

$$pH = -\log a_{H^+} = -\log(\gamma_{H^+} \cdot [H^+]) \quad \textbf{(S5)}$$

where γ_{H^+} is the activity coefficient for H^+, which was estimated using the Davies equation:

$$\log \gamma_{H^+} = -AZ_{H^+}^2 \left(\frac{\sqrt{I_m}}{1+\sqrt{I_m}} - 0.3I_m\right) \quad \textbf{(S6)}$$

In this expression, A is a pre-factor, Z_{H^+} is the valence of H^+ (= 1), and I_m the ionic strength of the electrolyte. The following expression was used to calculate A:

$$A = 1.82 \cdot 10^6 \cdot (\epsilon T)^{-\frac{3}{2}} \quad \textbf{(S7)}$$

where ϵ is the dielectric constant, which, in water, was approximated to 78.4, and T is the electrolyte temperature. The ionic strength I_m was calculated using the following expression:

$$I_m = \frac{1}{2}\sum Z_i^2 c_i \quad \textbf{(S8)}$$

In this expression, Z_i and c_i are the valence and concentration of ion i.

S3. Species Distribution Calculation

The two ionization constants of carbonic acid were written as:

$$K_{a1} = \frac{[H^+][HCO_3^-]}{[H_2CO_3]} \quad \textbf{(S9)}$$

$$K_{a2} = \frac{[H^+][CO_3^{2-}]}{[HCO_3^-]} \quad \textbf{(S10)}$$

The following mass balance expression can be written for carbon species:

$$M_0 = [H_2CO_3] + [HCO_3^-] + [CO_3^{2-}] \quad \textbf{(S11)}$$

In which M_0 is the total molar concentration of carbon. From **eq. S9**:

$$[HCO_3^-] = \frac{K_{a1}[H_2CO_3]}{[H^+]} \quad \textbf{(S12)}$$

Using **eq. S12** in **eq. S10**:

$$[CO_3^{2-}] = \frac{K_{a2}K_{a1}[H_2CO_3]}{[H^+]^2} \quad \textbf{(S13)}$$

Using **eq. S12** and **eq. S13** in **eq. S11**:

$$M_0 = [H_2CO_3] + \frac{K_{a1}[H_2CO_3]}{[H^+]} + \frac{K_{a2}K_{a1}[H_2CO_3]}{[H^+]^2} \quad \textbf{(S14)}$$

Solving **eq. S14** for the mole fraction of carbonic acid:

$$x_{H_2CO_3} = \frac{[H_2CO_3]}{M_0} = \frac{1}{1+\frac{K_{a1}}{[H^+]}+\frac{K_{a2}K_{a1}}{[H^+]^2}} \quad \textbf{(S15)}$$

Rewriting the above expression as a function of pH and pK:

$$x_{H_2CO_3} = \frac{[H_2CO_3]}{M_0} = \frac{1}{1+10^{pH-pK_{a1}}+10^{2pH-pK_{a1}-pK_{a2}}} \quad \textbf{(S16)}$$

Analogously, the expressions for the concentrations of bicarbonate and carbonate are:

$$x_{HCO_3^-} = \frac{10^{pH-pK_{a1}}}{1+10^{pH-pK_{a1}}+10^{2pH-pK_{a1}-pK_{a2}}} \quad \textbf{(S17)}$$

$$x_{CO_3^{2-}} = \frac{10^{2pH-pK_{a1}-pK_{a2}}}{1+10^{pH-pK_{a1}}+10^{2pH-pK_{a1}-pK_{a2}}} \quad \textbf{(S18)}$$

$x_{H_2CO_3}$, $x_{HCO_3^-}$ and $x_{CO_3^{2-}}$ were used to plot the species distribution in **Figure S5** using pK values from **Table 1**.

S4. Malachite Formation

Copper electrodes were reduced for 20 minutes at – 0.7 V prior to cathodic sweeps to remove copper oxide contamination and prevent the precipitation of $Cu_2(OH)_2CO_3$, an insoluble salt (malachite).[4] Precipitation of malachite was studied in the absence of this reductive pre-treatment step at 25 °C. Polarization of the electrode between 0.1 and –0.6 V resulted in decrease of the carbonate mole fraction and increase of the bicarbonate mole fraction (**Figure S8a**). The depletion of carbonate is indicative of the precipitation of copper-II ions as $Cu_2(OH)_2CO_3$. These copper ions are formed from a leaching phenomenon initiated upon reduction of surface copper oxides contaminants at low cathodic potentials.[4] Precipitation of malachite consumes hydroxide anions, thereby significantly decreasing surface pH (**Figure S8b**). The presence of malachite was confirmed by the emergence of a band centered at 1370 cm^{-1}, which is assigned to one of the carbonate-centered modes of $Cu_2(OH)_2CO_3$, [5] and persisted on the surface regardless of potential (**Figure S8c**). Electrode images collected after reaction revealed an exposed area with green coloration, which is the typical color of malachite crystals (**Figure S8d**).

S5. Electrode Characterization Before and After Reaction

SEM images of the electrode collected before reaction at 25 °C revealed a rough copper surface uniformly covered with metal islands with cross-sectional lengths as long as 5 μm (**Figure S9a**). This landscape was expected due to the oxidation step in 10% H_3PO_4 prior to reaction. By applying a 1.6 V potential to the electrode, copper is stripped from the surface, thereby leading to rougher topographIES. **Figure S10** shows a representative AFM profile of the copper surface. These profiles showed that the copper metal islands have an average 33.9 nm peak-to-peak distance with features reaching up to 243.5 nm height on top of a continuous copper underlayer. The mean (Ra) and root mean square (Rq) roughness of the electrodes were estimated as 63.8 nm and 75.8 nm, respectively. With EDS, we observed that carbon (12.3%) and oxygen (6.6%) were also detected on the surface, likely due to atmospheric contamination during sample transfer, but phosphorus was not detected since the electrode surface was thoroughly rinsed with Milli-Q water prior to analysis (**Figure S9c**). SEM images of the electrode after cathodic sweep between 0.1 and –0.6 V at 25 °C showed that the surface was much smoother post-reaction (**Figure S9b**). 20 μm long copper conglomerates were observed throughout the surface. This smoothening is expected upon polarization to negative potentials as previous studies have established that polycrystalline copper reconstructs into Cu(111) and Cu(100) (flat surfaces with terrace-like sites) under CO_2RR potentials.[6] Our previous study has also shown smoothening of thin-film copper electrodes at different temperatures between 25 and 75 °C.[7] Atomic percentages for both copper and carbon are practically the same before and after reaction (**Figure S9c**). There is an approximate 5% increase in the oxygen contribution, which is likely due to oxidation during sample transfer. Copper XPS spectra revealed two pronounced peaks at 952 and 932 eV, which are assigned to the two split components $2p_{1/2}$ and $2p_{3/2}$, respectively (**Figure S11a**). Together, they indicate that copper is

mostly present on the surface as copper metal (Cu^0). Weak satellite peaks between 946 and 940 eV indicate that there is also some Cu^{1+} on the surface. There is no observable difference between copper spectra before and after reaction. Two overlapping peaks at 532 and 530.5 eV are observed on the oxygen XPS spectra (**Figure S11b**). Typically, the O_{1s} binding energy of many compounds falls withing a very narrow range, but these two peaks are conventionally assigned to oxygen in metal hydroxide and a metal carbonate forms, respectively. The relative decrease in the hydroxide contribution after reaction makes sense considering that the sample before reaction had just been oxidized in phosphoric acid, and that, upon reduction in 1.0 M potassium bicarbonate, hydroxide would be less prevalent than carbonate as a ligand to reduced copper. Carbon XPS spectra revealed three convoluted peaks between 290 and 283 eV (**Figure S11c**). These peaks are associated to adventitious carbon present as O – C = O (288 eV), C – O – C (286 eV), and C – C (285 eV). As there is no perceptible difference between these peaks in the samples before and after reaction, their contributions are likely derived from atmospheric contamination, and not reaction relevant.

S6. Mass Transfer Calculations

The mass transfer coefficient was defined as:

$$k_i = \frac{\dot{n}_i}{A \cdot \Delta C_i} \quad \textbf{(S19)}$$

where $\dot{n}_i$ is the molar flow rate transporting species i (mol·s^{-1}), A is the cross-sectional mass transfer area (m^2), and ΔC_i is the concentration difference of species i driving mass transport (mol·m^{-3}). From Fick's first law, the molar flux J (mol·m^{-2}·s^{-1}) can be written as:

$$J_i = \frac{\dot{n}_i}{A} = -D_i \cdot \frac{dC_i}{dx} \quad \textbf{(S20)}$$

Discretizing for a concentration gradient across the boundary layer, **eq. S20** becomes:

$$\frac{\dot{n}_i}{A} = D_i \cdot \frac{\Delta C_i}{\sigma} \quad \textbf{(S21)}$$

where σ is the boundary layer thickness. Substituting the above expression into the first expression:

$$k_i = \frac{D_i}{\sigma} \quad \textbf{(S22)}$$

Mass transfer coefficients were calculated for the bicarbonate species using the experimentally determined boundary layer thickness and its diffusivity. The temperature dependence of diffusivity is widely calculated in the literature using the Stokes-Einstein equation for spherical species in liquid media:

$$D_i = \frac{k_B T}{6\pi\eta_i r_i} \quad \textbf{(S23)}$$

In this expression, k_B is the Boltzmann constant (J·K^{-1}), T is temperature (K), η is the liquid viscosity (Pa·s), which is also a function of temperature, and r is the radius of the spherical species (m). While the Stokes-Einstein relation does not directly apply to ionic solutes in aqueous electrolytes, the temperature-dependence implied by it seems to hold for major seawater ions,[8] and agree with diffusivities calculated by Zeebe via molecular dynamics simulations for CO_2,

bicarbonate and carbonate between 273 and 373 K.[9] The expression derived by Zeebe for bicarbonate diffusivity was used in this work:

$$D_{HCO_3^-} = 7.02 \cdot 10^{-9} \cdot \left(\frac{T}{204.03} - 1\right)^{2.39} \qquad \textbf{(S24)}$$

Sherwood numbers were calculated using **eq. S25**:

$$Sh = \frac{k_i L}{D_i} = \frac{L}{\sigma} \qquad \textbf{(S25)}$$

In this expression, L is the characteristic length of the system, which was taken as half the vertical distance of the free cell volume compartment, i.e. L = 1 cm.

Supporting Figures

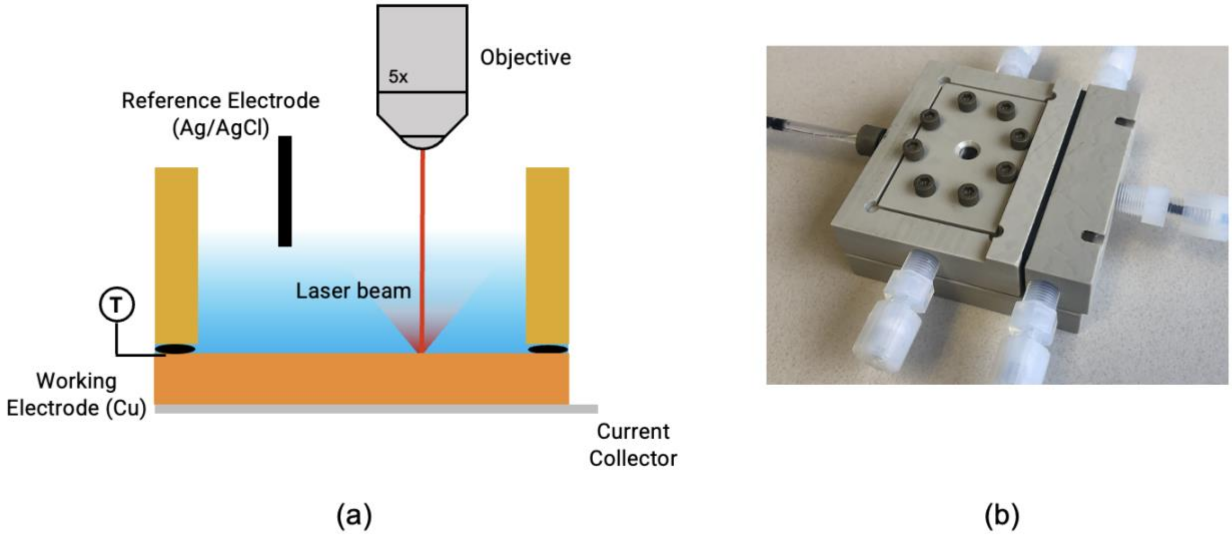

Figure S1. Experimental setup used for local pH measurements. (a) Schematic representation of the temperature-controlled cathodic compartment. (b) Two-compartment, three-electrode spectroelectrochemical cell.

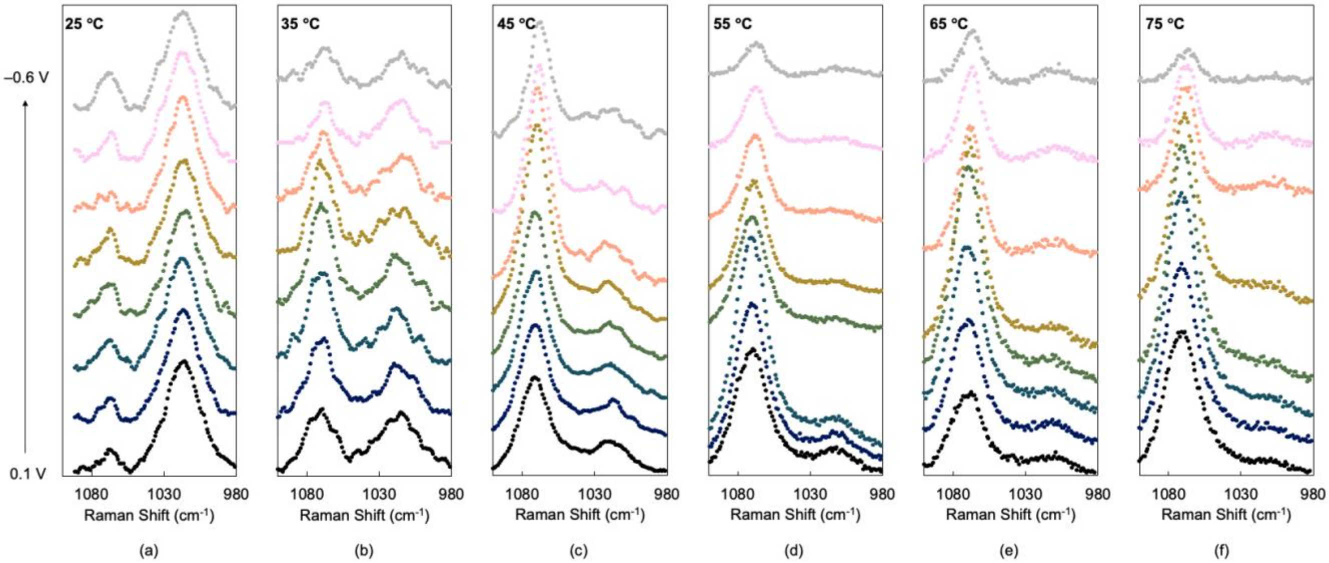

Figure S2. Raman spectra of bicarbonate and carbonate bands at (a) 25, (b) 35, (c) 45, (d), 55, (e) 65, and (f) 75 °C between 0.1 and –0.6 V vs. RHE.

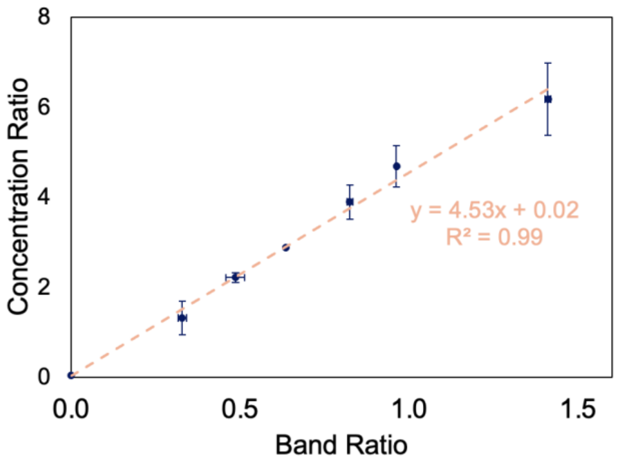

Figure S3. Experimental calibration curve correlating local concentration ratio and band ratio between bicarbonate and carbonate anions.

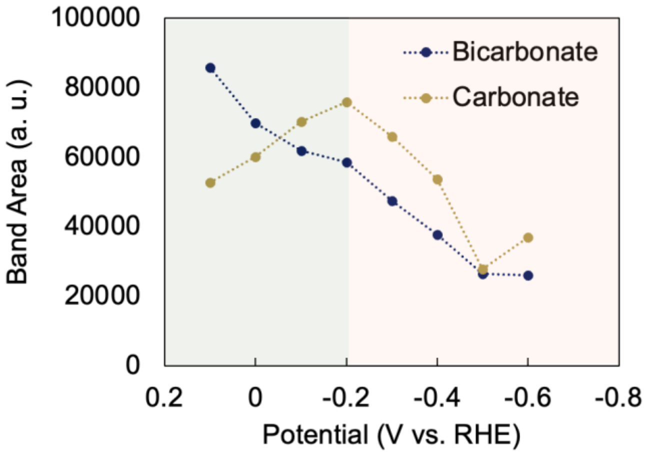

Figure S4. Absolute areas of bicarbonate and carbonate bands as a function of potential at 35 °C.

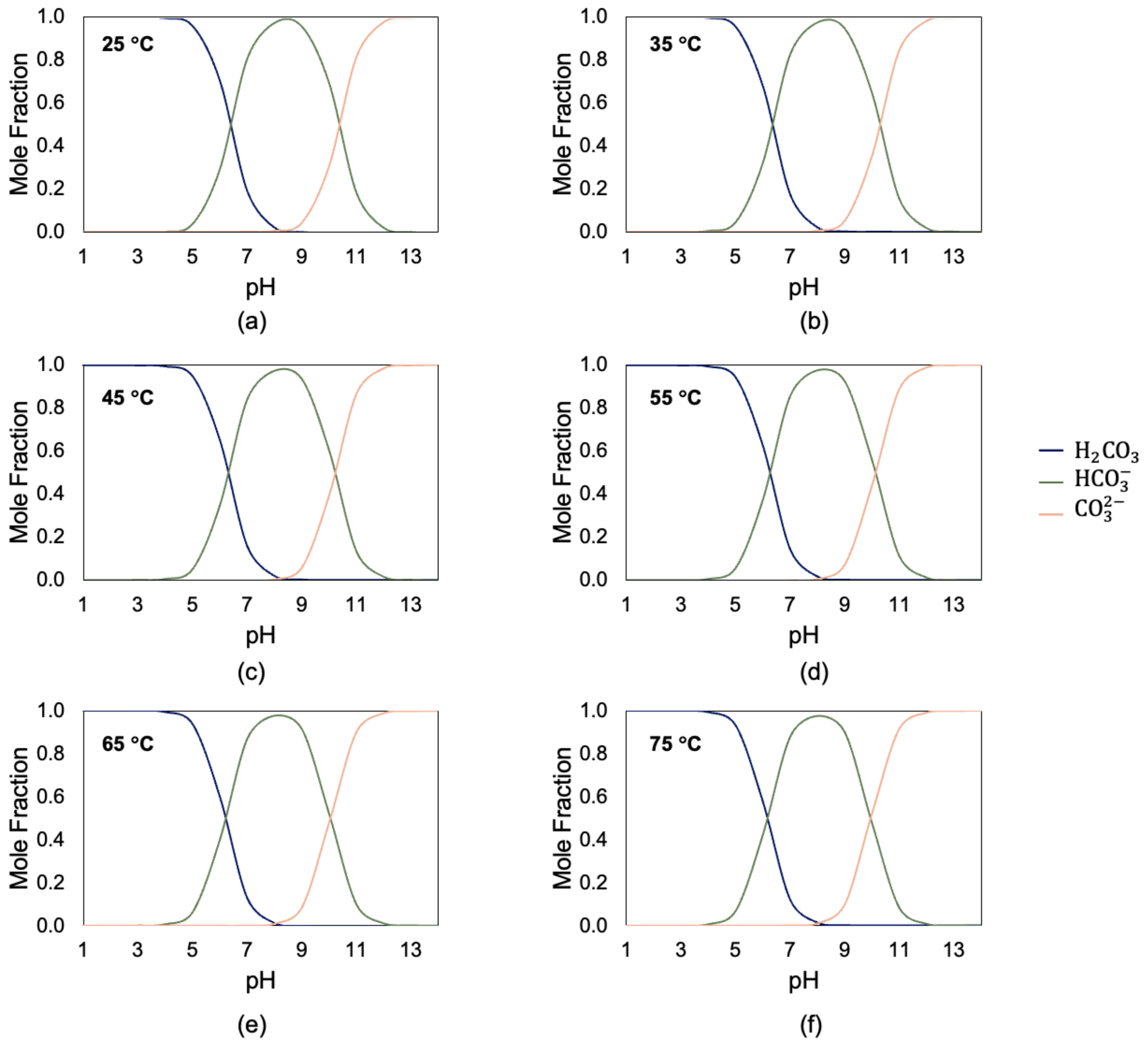

Figure S5. Species distribution for $H_2CO_3/HCO_3^-/CO_3^{2-}$ equilibrium between 25 and 75 °C (a-f). Increasing temperature favors the formation of carbonate at lower pH values (curves shift to the left), even though differences are minimal.

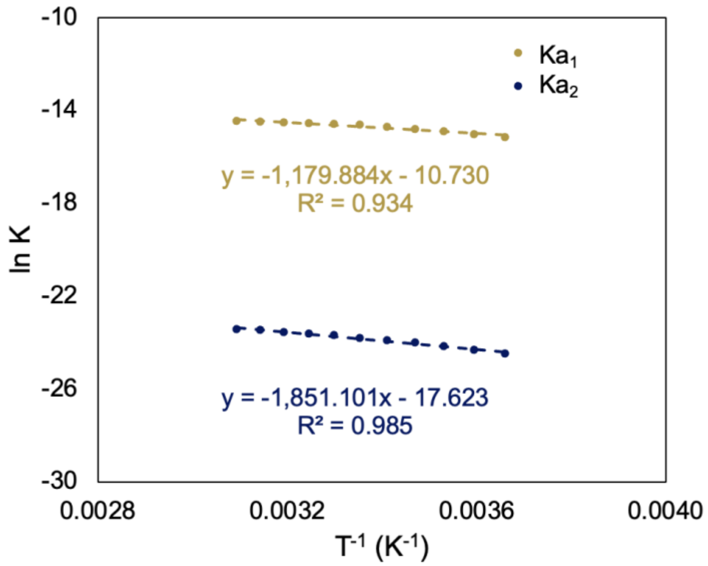

Figure S6. Van't Hoff plots for the first and second ionization constants of carbonic acid based on Harned et al.'s work between 0 and 50 °C.[2, 3]

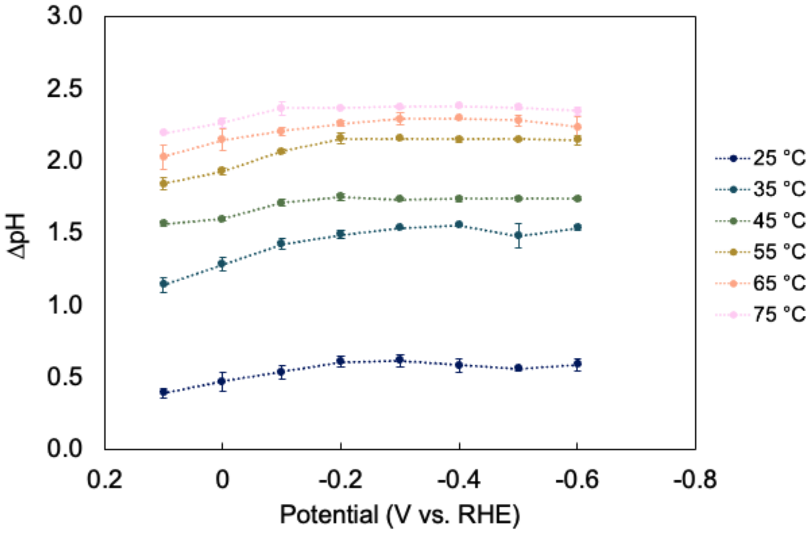

Figure S7. Difference between surface pH and electrolyte bulk pH (ΔpH) as function of potential and temperature. ΔpH increases with increasing temperature regardless of potential.

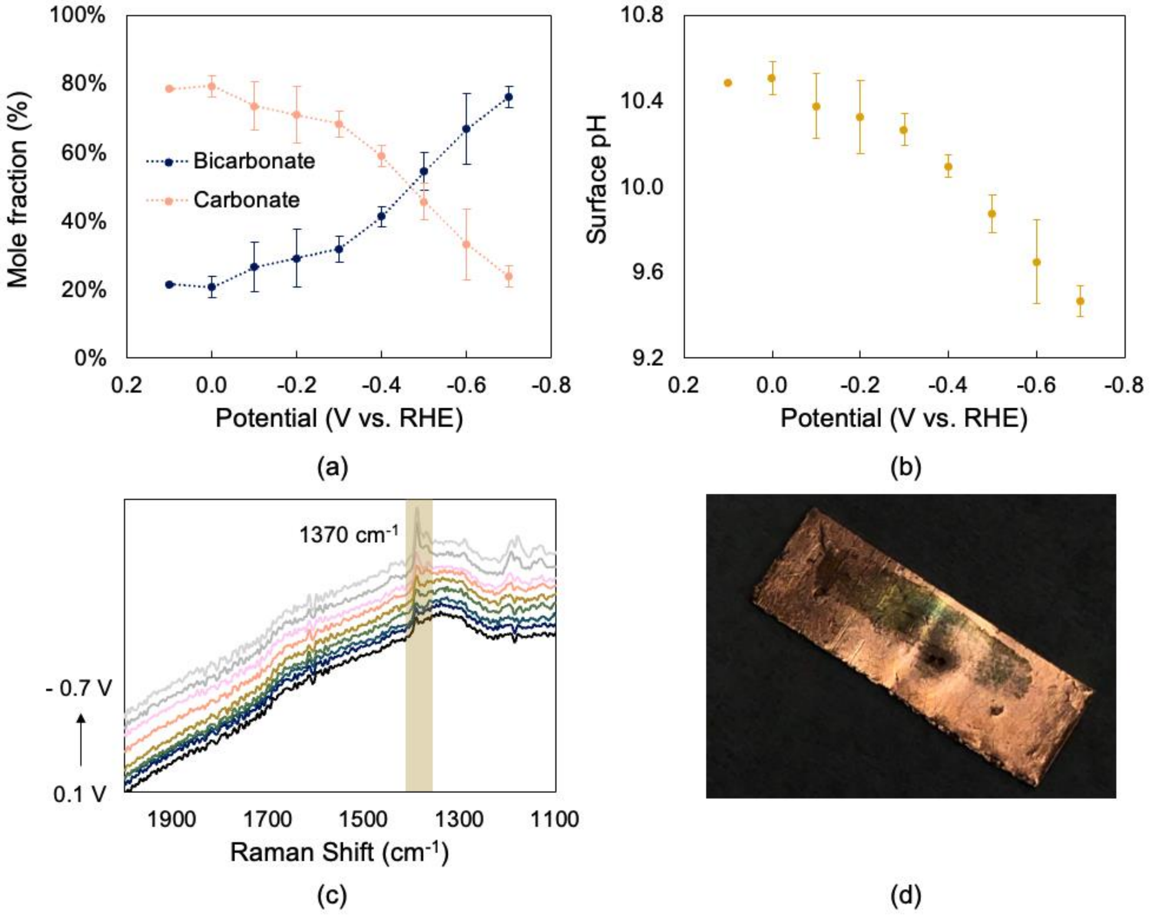

Figure S8. Evidence of $Cu_2(OH)_2CO_3$ formation in experiments without 20-minute reductive pre-treatment step. (a) Mole fractions of anions as a function of potential at 25 °C, revealing depletion of carbonate due to malachite formation. (b) Resulting decrease in surface pH due to consumption of hydroxide anions for malachite precipitation. (c) Representative spectra showing 1370 cm^{-1} band assigned to malachite. (d) Image of electrode after reaction revealing green-colored region.

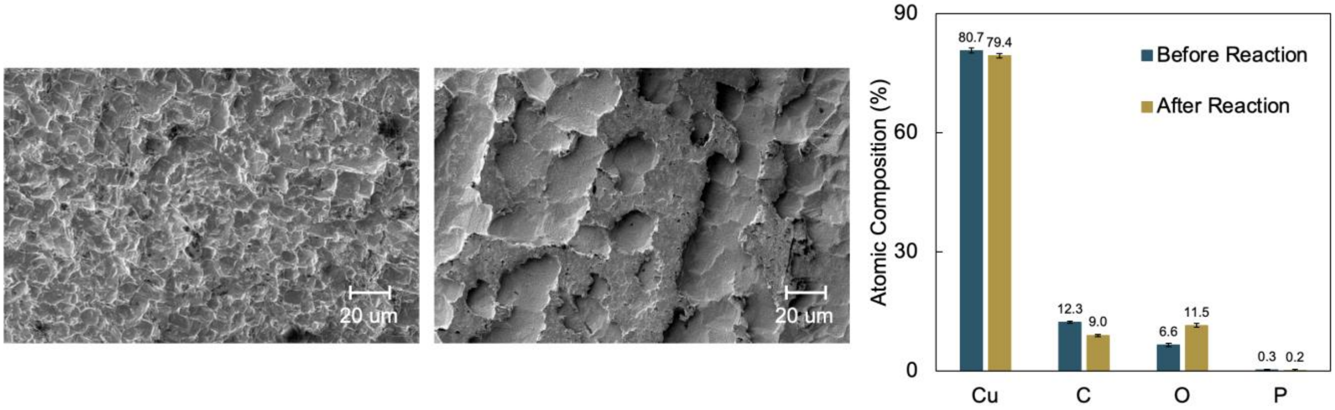

(a) (b) (c)

Figure S9. Electrode morphology probed with SEM/EDS. Images of electrode surface (a) before and (b) after reaction at 25 °C. (c) Surface atomic composition calculated from EDS spectra.

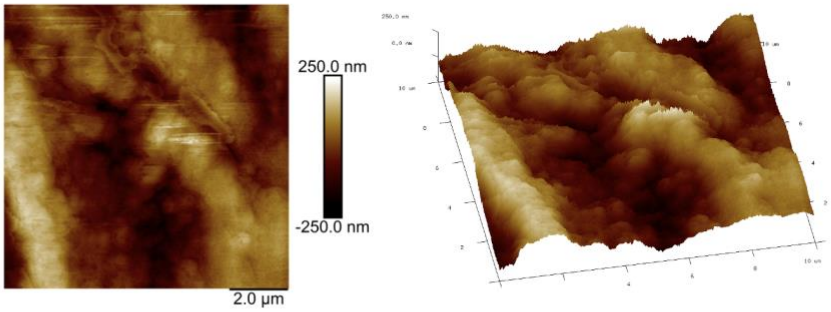

(a) (b)

Figure S10. Copper surface topography analysis before reaction at 25 °C. (a) AFM image taken with on a 10-µm scan size grid. (b) Associated tridimensional profile highlighting surface features.

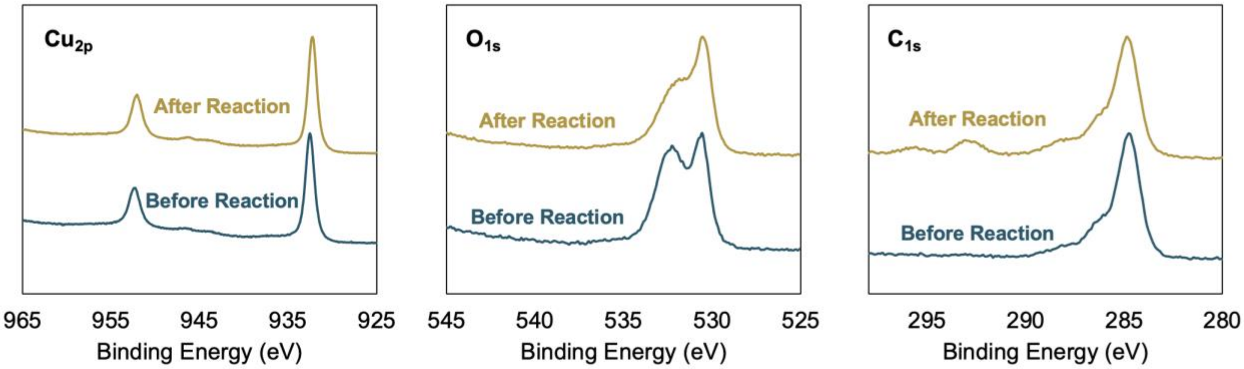

(a) (b) (c)

Figure S11. XPS spectra of copper electrode before and after reaction at 25 °C showing (a) Cu_{2p}; (b) O_{1s} and (c) C_{1s} lines.

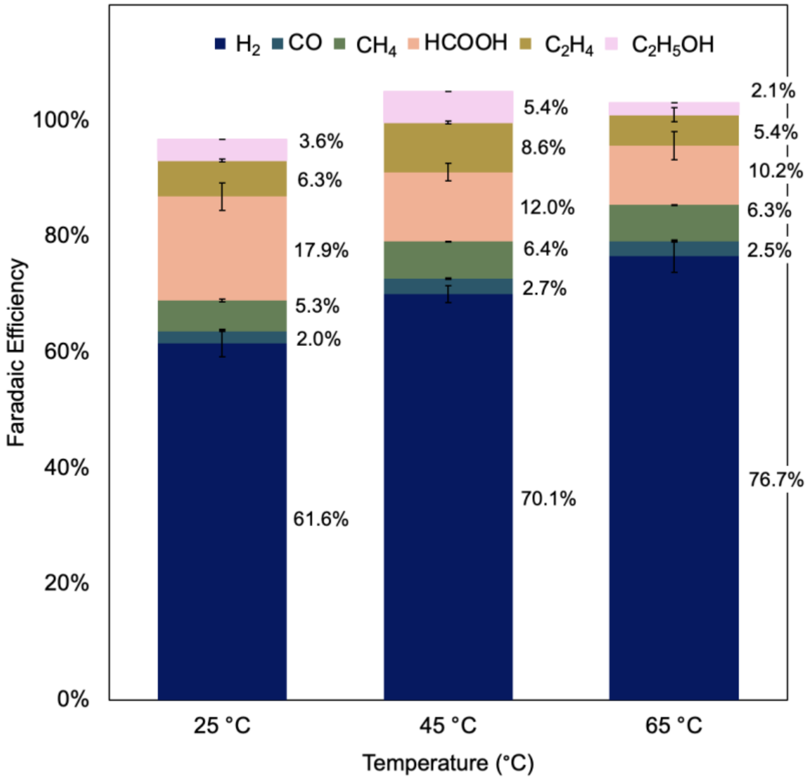

Figure S12. Individual Faradaic efficiencies for hydrogen, carbon monoxide, methane, ethylene, and formic acid major six products at 25, 45, and 65 °C at – 0.6 V (vs. RHE).

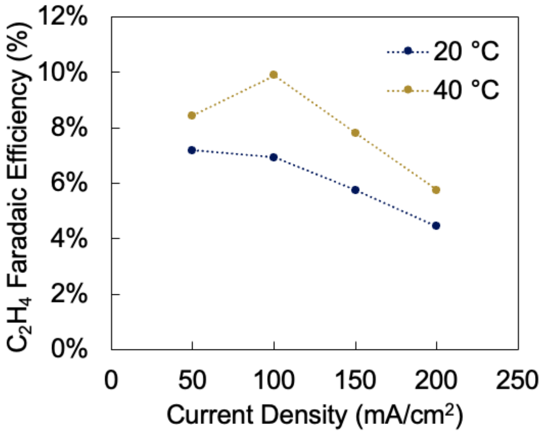

Figure S13. Faradaic efficiency of ethylene under high current density operation in MEA electrolyzer at 20 and 40 °C.

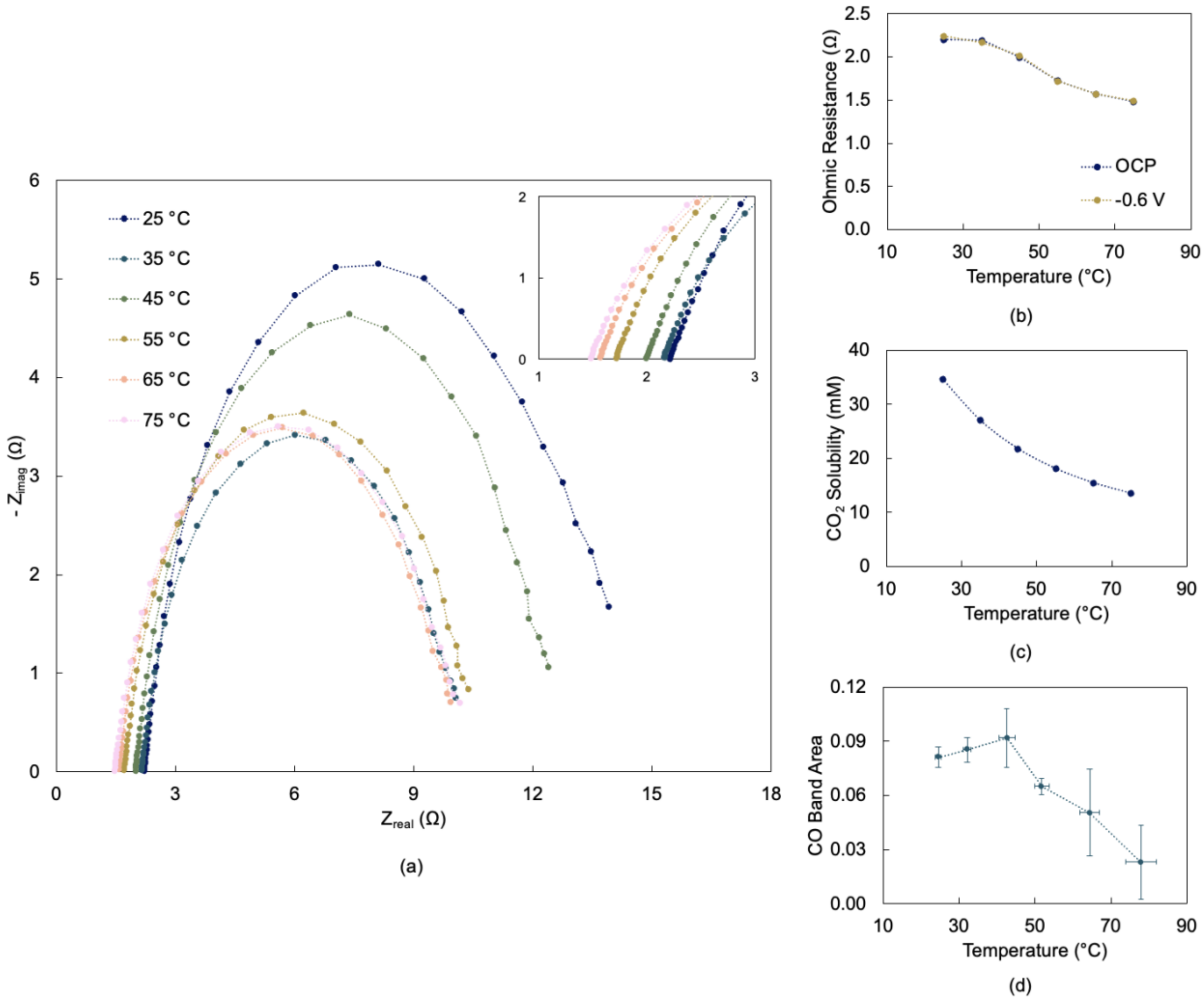

Figure S14. Effect of temperature on electrolyte resistance, CO_2 solubility and CO intermediate adsorption. (a) Electrochemical impedance spectroscopy (EIS) Nyquist plots collected at –0.6 V. Inset highlights the effect of higher temperatures on decreasing the electrolyte resistance. (b) Calculated electrolyte resistance as a function of temperature at –0.6 V and at open circuit potential (OCP). (c) CO_2 solubility as a function of temperature between 25 and 75 °C based on Carroll et al.'s correlation for Henry's constant.[10] (d) Linearly-adsorbed CO_{ad} area as a function of temperature showing CO coverage at – 0.6 V.

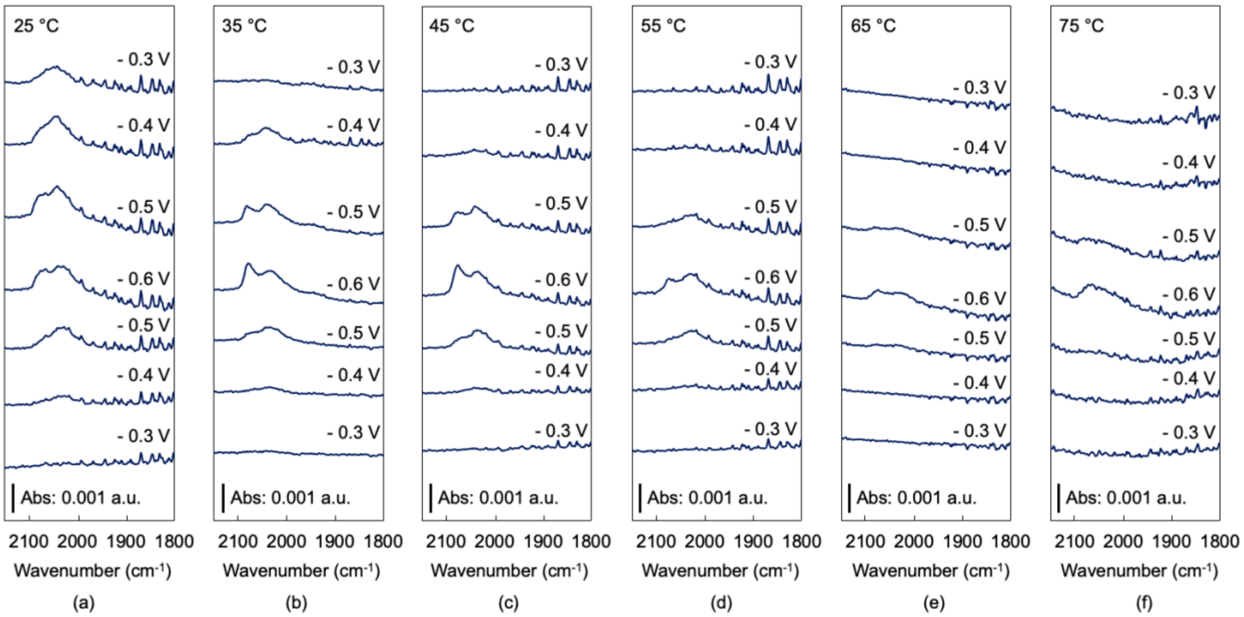

Figure S15. Infrared spectra of CO band between 2150 and 1800 cm^{-1} at (a) 25, (b) 35, (c) 45, (d), 55, (e) 65, and (f) 75 °C between –0.3 and –0.6 V vs. RHE. Linearly-bound CO was observed between 2100 and 2000 cm^{-1},with high- and low-frequency band contributions. Bridging CO was not observed between 1950 and 1800 cm^{-1}.

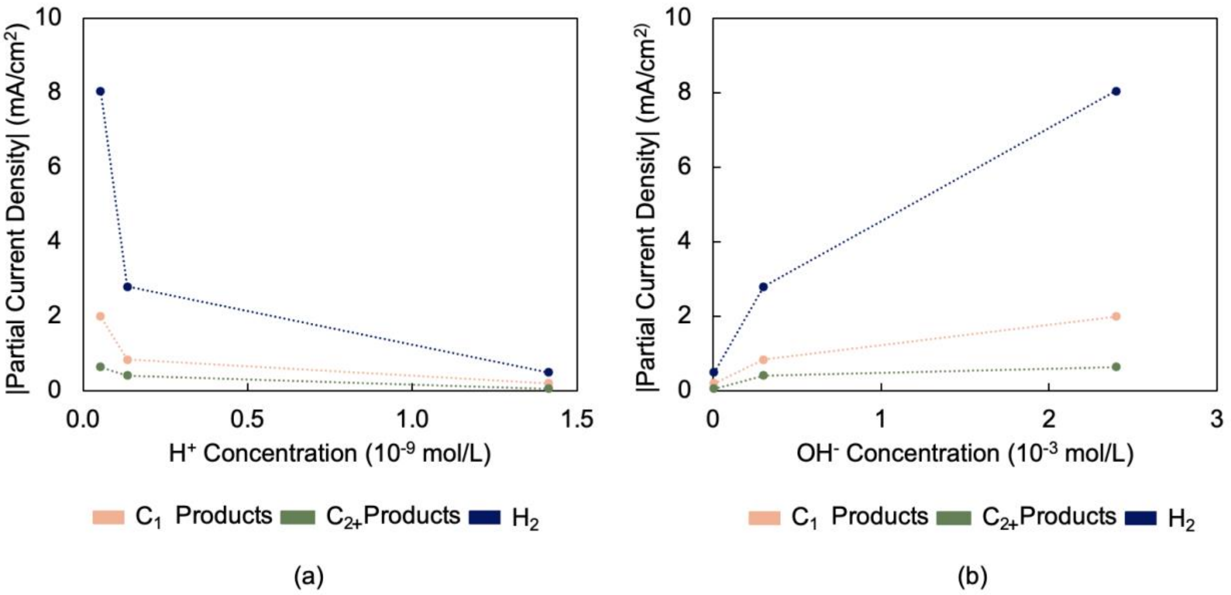

Figure S16. Product current densities at –0.6 V as a function of the concentration of (a) protons and (b) hydroxide anions.

Supporting Tables

Table S1. pK_{a1} and pK_{a2} of carbonic acid extrapolated from Harned et al.'s ionization constants between 0 and 50 °C.[2, 3] pK_w values taken from the Engineering Toolbox.[11]

Temperature (°C)	pK_{a1}	pK_{a2}	pK_w
25	6.379	10.350	13.995
35	6.323	10.263	13.680
45	6.271	10.181	13.396
55	6.222	10.104	13.140
65	6.176	10.031	12.907
75	6.132	9.963	12.696

Table S2. Electrolyte bulk pH at different temperatures.

Temperature (°C)	pH (theoretical)	pH (measured)
25	8.36	8.38
35	8.29	8.09
45	8.23	8.18
55	8.16	8.19
65	8.10	8.11
75	8.05	8.01

References

(1) Song, H.; Fernández, C. A.; Venkataraman, A.; Brandão, V. D.; Dhingra, S. S.; Arora, S. S.; Bhargava, S. S.; Villa, C. M.; Sievers, C.; Nair, S.; Hatzell, M. C. Ethylene Production from Carbonate Using a Bipolar Membrane Electrolysis System. *ACS Applied Energy Materials* **2024**, *7* (3), 1224-1233. DOI: 10.1021/acsaem.3c02758.

(2) Harned, H. S.; Davis, R., Jr. The Ionization Constant of Carbonic Acid in Water and the Solubility of Carbon Dioxide in Water and Aqueous Salt Solutions from 0 to 50°. *Journal of the American Chemical Society* **1943**, *65* (10), 2030-2037. DOI: 10.1021/ja01250a059.

(3) Harned, H. S.; Scholes, S. R., Jr. The Ionization Constant of HCO3- from 0 to 50°. *Journal of the American Chemical Society* **1941**, *63* (6), 1706-1709. DOI: 10.1021/ja01851a058.

(4) Henckel, D. A.; Counihan, M. J.; Holmes, H. E.; Chen, X.; Nwabara, U. O.; Verma, S.; Rodríguez-López, J.; Kenis, P. J. A.; Gewirth, A. A. Potential Dependence of the Local pH in a CO2 Reduction Electrolyzer. *ACS Catalysis* **2021**, *11* (1), 255-263. DOI: 10.1021/acscatal.0c04297.

(5) Frost, R. L.; Martens, W. N.; Rintoul, L.; Mahmutagic, E.; Kloprogge, J. T. Raman spectroscopic study of azurite and malachite at 298 and 77 K. *Journal of Raman Spectroscopy* **2002**, *33* (4), 252-259. DOI: https://doi.org/10.1002/jrs.848.

(6) Kim, Y.-G.; Baricuatro, J. H.; Javier, A.; Gregoire, J. M.; Soriaga, M. P. The Evolution of the Polycrystalline Copper Surface, First to Cu(111) and Then to Cu(100), at a Fixed CO2RR Potential: A Study by Operando EC-STM. *Langmuir* **2014**, *30* (50), 15053-15056. DOI: 10.1021/la504445g.

(7) Brandão, V. D.; Song, H.; Venkataraman, A.; Fishler, Y.; Arora, S. S.; Bhargava, S. S.; Villa, C.; Holewinski, A.; Nair, S.; Hatzell, M. C.; Sievers, C. Temperature Effects on the Surface CO Population during CO2 Electroreduction over Copper. *ACS Catalysis* **2025**, 8979-8990. DOI: 10.1021/acscatal.5c01173.
(8) Yuan-Hui, L.; Gregory, S. Diffusion of ions in sea water and in deep-sea sediments. *Geochimica et Cosmochimica Acta* **1974**, *38* (5), 703-714. DOI: https://doi.org/10.1016/0016-7037(74)90145-8.
(9) Zeebe, R. E. On the molecular diffusion coefficients of dissolved CO2,HCO3-, and CO32- and their dependence on isotopic mass. *Geochimica et Cosmochimica Acta* **2011**, *75* (9), 2483-2498. DOI: https://doi.org/10.1016/j.gca.2011.02.010.
(10) Carroll, J. J.; Slupsky, J. D.; Mather, A. E. The Solubility of Carbon Dioxide in Water at Low Pressure. *Journal of Physical and Chemical Reference Data* **1991**, *20* (6), 1201-1209. DOI: 10.1063/1.555900 (acccessed 11/11/2024).
(11) Water - Ionization Constant, pKw, of Normal and Heavy Water. *The Engineering ToolBox* **2017**.
